# Case Report: Prostate metastasis from pulmonary sarcomatoid carcinoma: the first reported case and clinical implications of palliative radiotherapy

**DOI:** 10.3389/fonc.2026.1949095

**Published:** 2026-08-27

**Authors:** Xueping Liu, Nai Mu, Guobo Du, Min Hou, Lang He

**Affiliations:** 1 Cancer prevention and treatment institute of Chengdu, Department of oncology, Chengdu Fifth People’s Hospital/The Second Clinical Medical College, Affiliated Fifth People’s Hospital of Chengdu University of Traditional Chinese Medicine, Chengdu, China; 2 Geriatric Diseases Institute of Chengdu, Department of Orthopedics, Chengdu Fifth People’s Hospital/The Second Clinical Medical College, Affiliated Fifth People’s Hospital of Chengdu University of Traditional Chinese Medicine, Chengdu, China; 3 Department of Medical Oncology, Affiliated Hospital of North Sichuan Medical College, Nanchong, Sichuan, China

**Keywords:** case report, differential diagnosis, immunohistochemistry, palliative radiotherapy, prostate metastasis, pulmonary sarcomatoid carcinoma

## Abstract

Pulmonary sarcomatoid carcinoma (PSC) is a highly aggressive lung cancer subtype. Prostate metastasis is extremely rare in lung cancer, and no PSC case with immunohistochemical confirmation has been reported. We report a 68-year-old man diagnosed with left upper lobe carcinosarcoma (myogenic differentiation) on pathological biopsy. He underwent multimodal treatment, including radical resection, adjuvant chemotherapy, anlotinib targeted therapy, and local thoracic radiotherapy. Eight months later, he developed progressive dysuria. Whole-body positron emission tomography-computed tomography (PET-CT) showed hypermetabolic lesions in the prostate and multiple systemic sites, while the serum prostate-specific antigen (PSA) level remained normal. Transurethral resection of the prostate (TURP) was performed, and pathology confirmed metastatic sarcomatoid carcinoma. Palliative radiotherapy to the prostate (62.5 Gy/25 fractions) completely resolved his urinary symptoms. The patient died 11 months after diagnosis from pulmonary infection. This is the first documented case of prostate metastasis from PSC worldwide. For such patients, histopathological and immunohistochemical confirmation is indispensable, and palliative radiotherapy can effectively relieve urinary symptoms.

## 1 Introduction

PSC is a rare histological variant of non-small cell lung cancer (NSCLC). It accounts for 0.3% to 3% of all primary malignant lung neoplasms. Carcinosarcoma is one of the five well-recognized subtypes of PSC. It is distinguished by a biphasic differentiation pattern, with both carcinomatous epithelial and sarcomatous mesenchymal components (1). The prognosis of PSC is extremely poor, with a median overall survival of only 6–12 months even after radical resection, due to its high invasiveness and early metastatic potential (2).

Prostate metastases from solid tumors are rare, accounting for <1% of all prostatic surgical specimens (3). Most secondary prostate tumors arise from direct invasion of bladder urothelial carcinoma, while distant metastases are uncommon. Lung cancer is a rare source of distant prostate metastasis, with reported cases limited to small cell lung cancer, adenocarcinoma, and large cell neuroendocrine carcinoma (LCNEC) (4–6). To date, no case of PSC metastasizing to the prostate has been documented in the English literature.

Here, we report the first case of prostate metastasis from PSC, and discuss its clinical characteristics, diagnostic approach and treatment strategies based on a systematic literature review.

## 2 Case report

**Figure 1:**
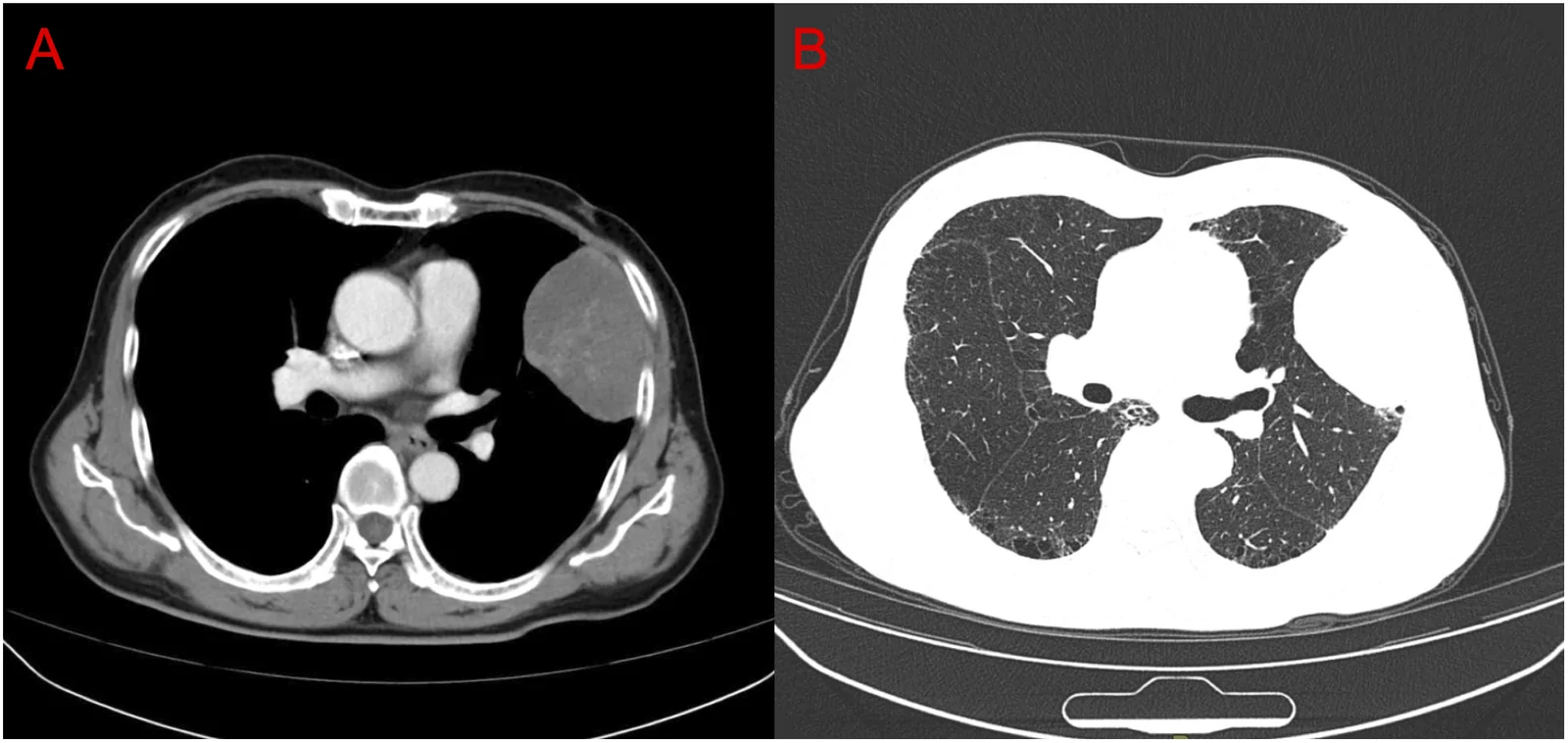
Chest enhanced CT demonstrated a heterogeneous mass in the left upper lobe before surgery. **(A)** Soft tissue window. **(B)** Lung window.

**Figure 2:**
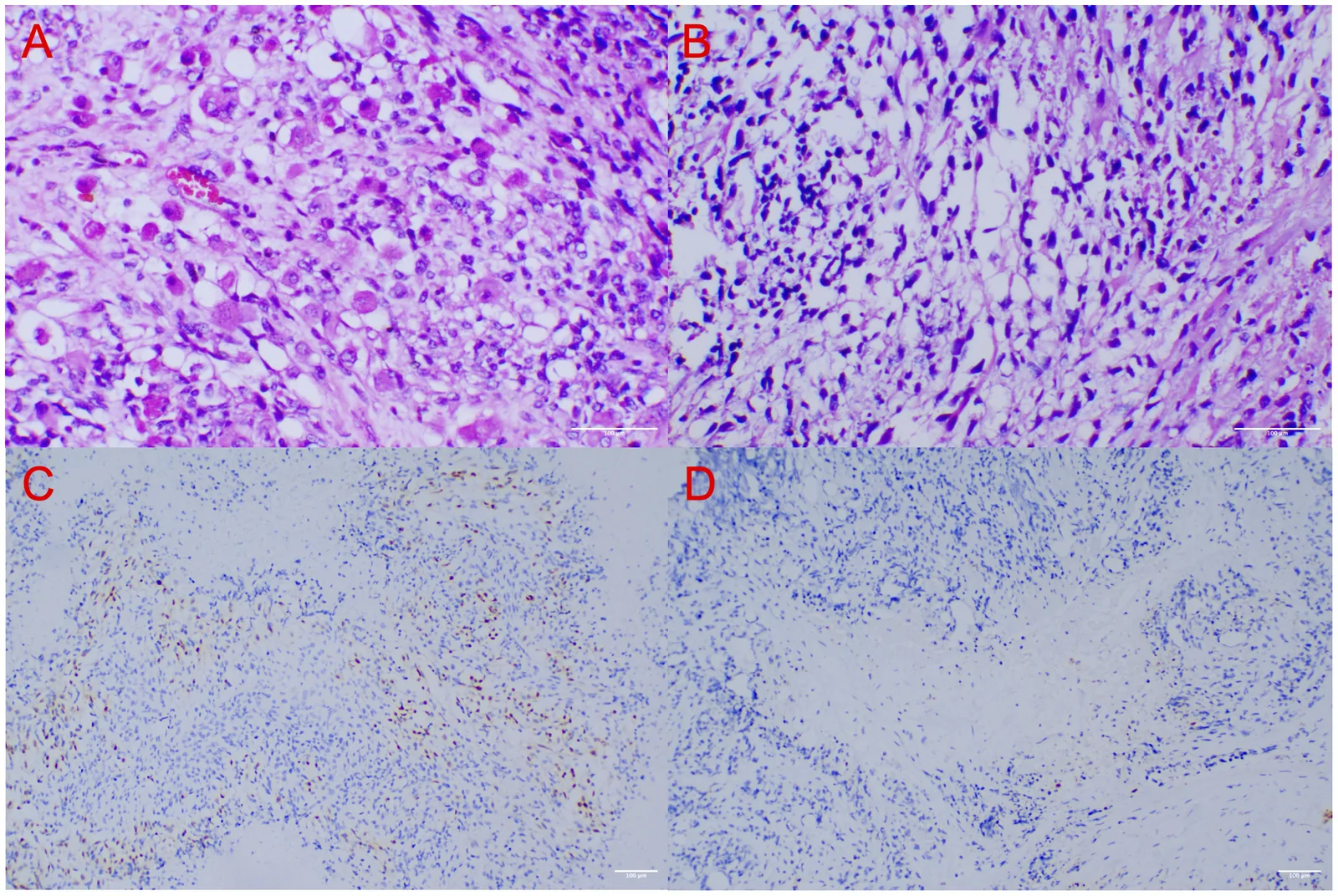
Histopathological and immunohistochemical features of primary pulmonary sarcomatoid carcinoma and prostatic metastasis. **(A)** Primary pulmonary tumor showing pleomorphic sarcomatous cells with nuclear atypia (H&E, ×200, scale bar = 100 μm). **(B)** Metastatic tumor in the prostate with identical spindle cell morphology (H&E, ×200, scale bar = 100 μm). **(C)** Positive immunohistochemical staining for myogenin in the metastatic lesion, confirming myogenic differentiation (×100, scale bar = 100 μm). **(D)** Negative immunohistochemical staining for PSA in the metastatic lesion, confirming non-prostatic origin (×100, scale bar = 100 μm).

A 68-year-old man with a 40-year smoking history presented with persistent cough in February 2023. Chest computed tomography (CT) revealed an 8.7 × 5.8 cm mass in the left upper lobe (**Figure 1**). He underwent thoracoscopic left upper lobectomy with mediastinal lymphadenectomy. Postoperative pathology confirmed carcinosarcoma with both carcinomatous epithelial and sarcomatous mesenchymal components, with no lymph node involvement (0/12). The epithelial component showed poorly differentiated carcinoma, while the sarcomatous component exhibited myogenic differentiation. Immunohistochemistry (IHC) showed CK (partial +), Desmin (+), myogenin (partial +), Ki-67 (hot spot 60%), TTF1 (−), CK7 (−) (**Figure 2A**).

Postoperatively, the patient received three cycles of adjuvant AI chemotherapy (90 mg/m² epirubicin plus 1.25 g/m² ifosfamide), followed by radiotherapy to the left lung (60 Gy/24 fractions) and continuous oral anlotinib (10 mg daily).

**Figure 3:**
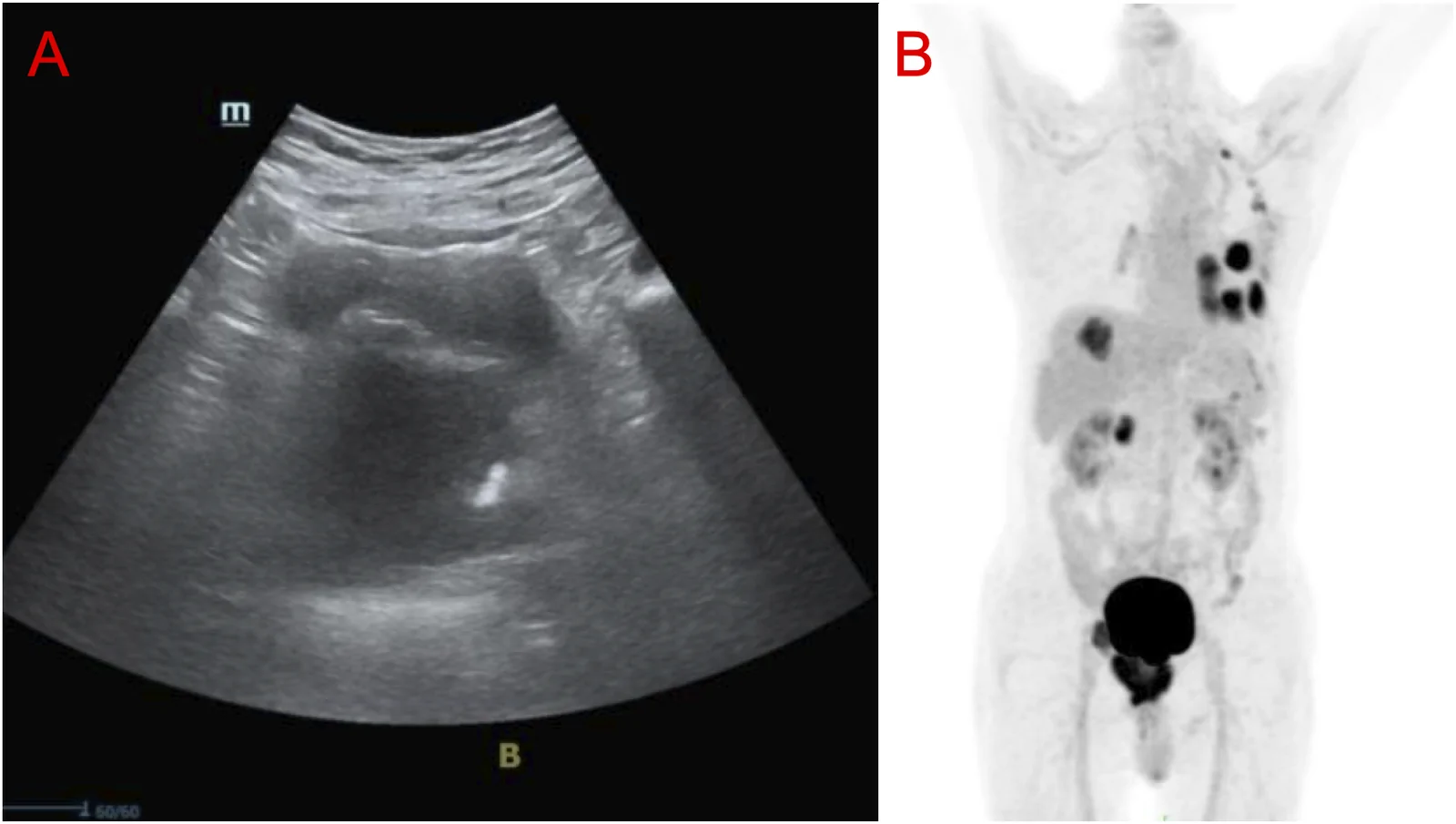
Pelvic ultrasound and whole-body PET-CT showing prostatic enlargement and widespread metastatic disease. **(A)** Transrectal ultrasound showing a 6.0 × 7.0 × 5.5 cm prostate mass with heterogeneous echotexture. **(B)** Whole-body PET-CT demonstrating widespread metastatic lesions involving the pleura, seminal vesicle, pelvis, liver, pancreas, and prostate.

In October 2023, he developed progressive dysuria. Serum total PSA was within normal range (0.21 ng/mL). Transrectal ultrasound showed a 6.0 × 7.0 × 5.5 cm prostate mass with heterogeneous echotexture (**Figure 3A**). TURP was performed, and pathology showed infiltration by atypical spindle cells. IHC of the prostate lesion: CK (positive in rare tumor cells), Vimentin (+), MyoD1 (+), TLE-1 (+), Ki-67 (hot spot 50%), myogenin (focal weak +), PSA (−), consistent with sarcomatoid carcinoma metastasis (**Figures 2B–D**).

Whole-body PET-CT demonstrated widespread metastases involving the left pleura, right seminal vesicle, right pelvis, liver, pancreas and prostate (**Figure 3B**). After multidisciplinary discussion, palliative radiotherapy to the prostate (62.5 Gy/25 fractions) was initiated. Urinary symptoms improved significantly after 15 fractions and completely resolved at the end of radiotherapy.

In January 2024, the patient was admitted with dyspnea due to massive left pleural effusion and atelectasis. Secondary pulmonary infection developed as a complication of the malignant pleural effusion. Despite supportive care, he died of this tumor-associated pulmonary infection, with an overall survival of 11 months.

## 3 Discussion

PSC is a highly aggressive tumor. It mainly spreads through the bloodstream to the brain, bone and liver, and its metastatic behavior is closely linked to epithelial-mesenchymal transition (EMT). Prostate metastasis from PSC, however, is extremely rare. Three factors may account for this rarity: the relatively low blood supply of the prostate, shunting of tumor cells to the spine via the Batson vertebral venous plexus, and the lack of compatible homing signals for PSC cells in the prostate microenvironment.

We systematically reviewed 13 pathologically confirmed cases of lung cancer metastasizing to the prostate, published between 1983 and 2026 (4–16). As summarized in **Table 1**, adenocarcinoma was the most common primary histological subtype (8 cases), followed by small cell lung cancer (3 cases), large cell neuroendocrine carcinoma (1 case) and adenosquamous carcinoma (1 case). No previous study has reported prostate metastasis from pulmonary sarcomatoid carcinoma.

**Table 1:** Clinical characteristics and treatment outcomes of 14 cases with prostate metastasis from lung cancer (13 published cases plus the present case).

Characteristics	Total cohort (n = 14)
Age (years), median (range)	68 (23–82)
Male, n (%)	14 (100)
Histological subtype, n (%)	
Adenocarcinoma	8 (57.1)
Small cell lung cancer	3 (21.4)
Large cell neuroendocrine carcinoma	1 (7.1)
Adenosquamous carcinoma	1 (7.1)
Pulmonary sarcomatoid carcinoma (carcinosarcoma)	1 (7.1)
IHC profile, n (%)	
IHC data available	10 (71.4)
PSA negative (among tested)	10/10 (100)
TTF-1 positive (among adenocarcinoma tested)	6/7 (85.7)
Neuroendocrine markers positive (SCLC/LCNEC, among tested)	4/4 (100)
Time to prostate metastasis (months), median (range)	8 (0–36)
PSA level, n (%)	
Normal (≤ 4 ng/mL)	11 (78.6)
Elevated (> 4 ng/mL)	3 (21.4)
Metastasis extent, n (%)	
Widespread metastasis	12 (85.7)
Isolated/oligometastasis	2 (14.3)
Primary lung treatment, n (%)	
Systemic chemotherapy	8 (57.1)
Surgical resection	4 (28.6)
Thoracic radiotherapy	5 (35.7)
Targeted therapy/immunotherapy	3 (21.4)
Not specified	3 (21.4)
Local treatment for prostate, n (%)	
Palliative radiotherapy	4 (28.6)
TURP	5 (35.7)
No local treatment	5 (35.7)
Urinary symptom response (evaluable cases, n = 9)	
Complete/obvious relief	5
Partial relief	4
No relief	0
Survival after prostate metastasis (months), median (range)	4 (1–12+) [NR in 6 cases]
Overall survival (months), median (range)	11 (6 days–36+)
Present case	11

This case fills a gap in the metastatic spectrum of PSC in the literature. It also delivers an important clinical message: for patients with a history of lung cancer, even though the prostate is not a common metastatic site, new-onset urinary symptoms should trigger suspicion of prostate metastasis, especially in cases with aggressive histological subtypes.

Another key clinical takeaway is that primary prostate cancer and secondary prostatic metastases present nearly identical clinical features, with dysuria as the most common initial symptom. Although serum PSA is widely used as a routine diagnostic reference, PSA levels may remain within the normal range in patients with metastatic tumors involving the prostate, as these non-prostatic malignancies typically do not express androgen receptor or prostate-specific markers (17). This is particularly applicable to pulmonary sarcomatoid carcinoma, which lacks androgen receptor expression, thereby explaining the normal PSA level (0.21 ng/mL) observed in our patient. Therefore, PSA levels cannot reliably distinguish primary prostate cancer from metastatic involvement, and a definitive diagnosis can only be established by histopathological examination combined with immunohistochemical staining.

Primary prostatic adenocarcinoma typically expresses prostate-specific markers including PSA, prostate-specific membrane antigen (PSMA), and so on. Metastatic lesions from other primary sites, by contrast, show a biomarker expression pattern matching their original tumor. In our patient, the immunohistochemical profile of the prostatic metastasis was consistent with the primary PSC, with positive mesenchymal markers and negative PSA staining, which confirmed its pulmonary origin.

Pathological biopsy outcomes guide distinct therapeutic strategies for such patients. For primary prostatic tumors, androgen deprivation therapy is the core systemic treatment, supplemented by appropriate local therapy. For patients with prostate metastasis from lung cancer, however, systemic therapy for the primary tumor remains the foundation of management. Local treatment only serves palliative symptom control purposes, and more invasive radical prostatectomy should be avoided.

In our cohort, 9 patients received local therapy, and all evaluable cases experienced varying degrees of improvement in urinary symptoms. In this case, the patient had good performance status at baseline and preferred durable local tumor control. His urinary obstruction was manageable with spasmolytic and supportive care, with no indication for urgent decompression, which allowed delivery of conventionally fractionated radiotherapy. Given the relative radioresistance of the sarcomatoid component in PSC, we selected the 62.5 Gy/25-fraction regimen to elevate the biologically effective dose for improved local control. The patient’s urinary symptoms completely resolved by the end of radiotherapy, and no severe adverse events, including radiation cystitis or proctitis, were observed during treatment and follow-up, supporting the safety and preliminary efficacy of this high-dose palliative strategy. Unfortunately, the patient’s subsequent death from tumor-related pulmonary infection precluded long-term assessment of local control durability. Therefore, this regimen cannot be directly generalized to all patients.

Limitations of this case also include the absence of paired next-generation sequencing for both the primary lung tumor and metastatic prostate lesion. Definitive molecular confirmation of metastatic origin and underlying molecular pathways could therefore not be established. In addition, this is a single-case report, and the generalizability of our observations needs validation in larger patient cohorts.

## 4 Conclusion

This is the first documented case of prostate metastasis from PSC. Clinicians should suspect prostate metastasis in patients with a history of PSC who present with urinary symptoms, even with a normal PSA level. IHC is critical for accurate diagnosis, and palliative radiotherapy to the prostate provides effective symptomatic relief and should be included in multidisciplinary management.

## Data Availability

The datasets presented in this article are not readily available because No dedicated research dataset was generated for this single clinical case report, so no additional data access restrictions are applicable. All relevant clinical information has been fully presented in the article with patient privacy strictly protected. Requests to access the datasets should be directed to XL, 406463926@qq.com.
